# Efflux Pump Blockers in Gram-Negative Bacteria: The New Generation of Hydantoin Based-Modulators to Improve Antibiotic Activity

**DOI:** 10.3389/fmicb.2016.00622

**Published:** 2016-05-03

**Authors:** Ewa Otręebska-Machaj, Jacqueline Chevalier, Jadwiga Handzlik, Ewa Szymańska, Jakub Schabikowski, Gérard Boyer, Jean-Michel Bolla, Katarzyna Kieć-Kononowicz, Jean-Marie Pagès, Sandrine Alibert

**Affiliations:** ^1^Department of Technology and Biotechnology of Drugs, Medical College, Jagiellonian UniversityKrakow, Poland; ^2^UMR-MD1, Aix Marseille Université/IRBA, Facultés de Médecine et de PharmacieMarseille, France

**Keywords:** antibiotics, AcrAB pump, efflux pump blocker, hydantoin, multidrug resistance

## Abstract

Multidrug resistant (MDR) bacteria are an increasing health problem with the shortage of new active antibiotic agents. Among effective mechanisms that contribute to the spread of MDR Gram-negative bacteria are drug efflux pumps that expel clinically important antibiotic classes out of the cell. Drug pumps are attractive targets to restore the susceptibility toward the expelled antibiotics by impairing their efflux activity. Arylhydantoin derivatives were investigated for their potentiation of activities of selected antibiotics described as efflux substrates in *Enterobacter aerogenes* expressing or not AcrAB pump. Several compounds increased the bacterial susceptibility toward nalidixic acid, chloramphenicol and sparfloxacin and were further pharmacomodulated to obtain a better activity against the AcrAB producing bacteria.

## Introduction

Multidrug resistance (MDR) described in Gram-negative bacteria is continuously emerging as a prominent worldwide health concern (Chopra et al., 2008; Laxminarayan et al., 2008; Gandhi et al., 2010). One of the most contributing mechanisms is the overexpression of efflux pumps that are involved in bacterial survival, colonization and virulence (Delmar et al., 2014; Blair et al., 2015; Li et al., 2015; Venter et al., 2015; Davin-Regli et al., 2016). Several efflux pump superfamilies, e.g., major-facilitator (MF), multi-drug and toxic efflux (MATE), ATP-binding cassette (ABC), small multidrug resistance (SMR), resistance-nodulation-division (RND) transporters have been classified^1^,^2^ and extensively described in well-documented reviews: they differ by their functional structure and organization, their subcellular location inside the bacterium, their energy source (e.g., membrane potential for RND or ATP for ABC) and the involvement of a coupled antiport during the antibiotic expulsion (e.g., proton for AcrAB pump; Delmar et al., 2014; Blair et al., 2015; Li et al., 2015). The overexpression of Gram-negative efflux pumps, especially those belonging to the RND family, is now well-described in resistant isolates (Nikaido and Pagès, 2012; Li et al., 2015). This contributes to the acquisition of additional mechanisms of resistance including the mutation in antibiotic targets (e.g., mutation in gyrase/topoisomerase for quinolone) or the production of enzymes that degrade antibiotics (e.g., β-lactamases) and this can be associated or not with the alteration of the outer membrane permeability (Davin-Regli et al., 2008; Pagès et al., 2008). Regarding resistant clinical isolates of Gram-negative bacteria, the archetype of the drug active transporter system is the AcrAB-TolC/MexAB-OprM efflux pumps (Nikaido and Pagès, 2012; Li et al., 2015). The structures of components of efflux systems belonging to RND group, have been solved by X-ray crystallography and models of the pump assembly have been obtained (Yao et al., 2010; Du et al., 2014). The structure and function of the RND efflux pumps must be molecularly deciphered thus allowing the rational design and the synthesis of new compounds to combat MDR. The broad selectivity of efflux pumps makes difficult the identification of precise pharmacophoric groups at the drug surface. However, efflux pumps are attractive target by blocking this efflux mechanism in order to restore the intracellular concentration of antibacterial agents (Bolla et al., 2011; Ruggerone et al., 2013; Dreier and Ruggerone, 2015; Opperman and Nguyen, 2015). Recent computer docking analyses have produced some information about the involvement of certain amino acid residues, but clearly more chemical and biological information are needed to improve models (Schulz et al., 2010; Fischer et al., 2014). This is a ‘key’ point not only regarding the mode of action and dynamics of the process but also regarding the clinical impact of the design of new antibacterial agents: this last aspect is illustrated by the β-lactamases inhibitors currently used today (Bolla et al., 2011; Chen et al., 2013; Pucci and Bush, 2013).

To enhance the activity of old antibiotics by targeting resistance mechanisms in clinical resistant isolates, they can be combined with adjuvant molecules such as chemosensitizers (e.g., membrane permeabilizer or efflux inhibitor; Jones, 2010; Bolla et al., 2011). In addition, these types of transporter inhibitors may impair the activity of efflux pumps and thus reduce bacterial colonization and virulence (Venter et al., 2015; Davin-Regli et al., 2016). It must be noted that the efficacy of the inhibitors depends on their affinity for transporter binding sites (compared to the antibiotic) and their internal concentration close to the efflux pump. Consequently, due to these parameters associated with penetration rate and affinity for pump sites, some discrepancies can be observed in the level of the internally accumulated antibiotics depending on the bacterial backgrounds (Kaščáková et al., 2012; Cinquin et al., 2015).

Recently, two generations of hydantoin derivatives have been identified as AcrAB-TolC inhibitors with the *Enterobacter aerogenes* CM 64 strain that overproduces AcrAB (Handzlik et al., 2011). Compounds showing chemosensitizing effect on nalidixic acid activity were the starting point for new pharmacomodulations carried out in this study to obtain a new generation of products with an improved activity. Moreover, tests were extended to other chemically unrelated antibiotics, chloramphenicol and sparfloxacin, for which the antibacterial activity decreased together with the emergence of multidrug-resistant strains. Several chemical derivatives were synthesized to define pharmacophoric groups important for restoring the activity of antibiotics in AcrAB active bacteria.

## Materials and Methods

### Bacterial Strains

All generations of hydantoin derivatives were tested against two strains of *E. aerogenes*: the reference strain, ATCC 13048 (basal efflux producer) and its derivative strain, CM 64, a chloramphenicol-selected resistant strain that over-produces the AcrAB-TolC EPs (Ghisalberti et al., 2005). In addition, two isogenic strains EA289 (a clinical MDR strain that overproduced the AcrAB pump) and its EA294 derivative (an acr*AB* knockout strain) were used (Pradel and Pagès, 2002). These two strains contain additional mechanisms of antibiotic resistance such as ß-lactamases, targets mutations, etc. (Malléa et al., 1998; Pradel and Pagès, 2002; Kaščáková et al., 2012).

### Chemicals

**FIGURE 1:**
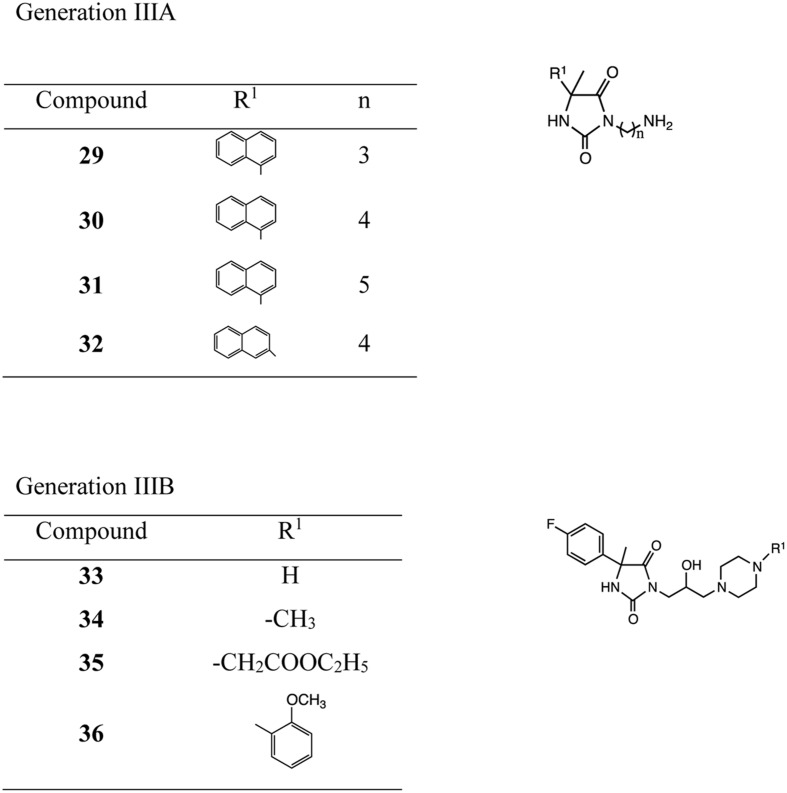
**Compounds of generation IIIA and generation IIIB of hydantoins**.

Hydantoin derivatives used in the pharmacological assays were obtained by chemical synthesis. The new generations IIIA and B were obtained using 3–4-step synthesis (Handzlik et al., 2014; Matys et al., 2015; **Figure 1** and Supplementary data). Purity and identity of new compounds were confirmed using spectral analysis (H-NMR, IR), elemental analysis and melting point measurements. Phenylalanine-Arginine β-naphthylamide (PAβN, dihydrochloride, Sigma) previously described as efflux pump inhibitor was used as reference (Bolla et al., 2011; Misra et al., 2015).

### Compound Susceptibility Assays

Susceptibilities of ATCC 13048, CM 64, EA294, and EA289 were determined by using the twofold standard microbroth dilution method (microplates and automatic analyses Tecan^®^; CLSI^3^). Approximately, 10^5^ CFU (colony forming unit) were inoculated in 200 μl of Mueller-Hinton II broth (MH II broth cation adjusted, Becton, Dickinson & Company) containing twofold serial dilutions of the targeted molecule. Experiments were performed in triplicate for each compound and each antibiotic. Results were estimated visually after 18 h incubation at 37°C (Philippe et al., 2015).

### Antibiotic Susceptibility Potentiating Assays

To assay the possible chemosensitizing activity of compounds, serial dilutions of antibiotics, nalidixic acid (NAL, Sigma), chloramphenicol (CHL, Sigma), doxycycline (DOX, hyclate, Sigma), erythromycin (ERY, lactobionate, AMDIPHARM) and sparfloxacin (SPX, Sigma), were incubated in the absence or in the presence of compounds. The antibiotics, NAL, CHL, and SPX are substrates of the AcrAB-TolC efflux pump as demonstrated by the increased MIC values obtained in CM 64 strain overexpressing the AcrAB-TolC pump (**Table 1**) compared to the reference one ATCC 13048. Thus we are able to hypothesize that an efflux blocker may reduce the antibiotic MIC in efflux producer strain. Generations IIIA and IIIB of hydantoin derivatives were tested at a concentration of 0.5 mM according to the intrinsic antibacterial activity of each compound (corresponding to the value of MIC/4). To facilitate the comparison of activity and the performance of a rational SAR analysis, they were additionally tested at the concentration corresponding to that of the best first generation of chemosensitizers (0.0625 mM; Handzlik et al., 2011). PAβN, the reference inhibitor for AcrAB pump, was used at 0.050 mM. Control experiments were carried out without compounds. Experiments were performed in triplicate for each antibiotic, each strain and each condition (without and with compound). The results were assessed after 18 h at 37°C and were presented by using the activity gain parameter A, the ratio of the MIC of the antibiotic (determined in the absence of compound) to its MIC in the presence of the compound.

**Table 1:** Susceptibility (MIC) of the *Enterobacter aerogenes* reference strain ATCC 13048, the derivative strain CM 64 overexpressing AcrAB-TolC pump, Ea289 overproducing the AcrAB pump and its derivative Ea294 (an acr*AB* knockout strain) to the different compounds belonging to various generations of hydantoins and to nalidixic acid (NAL), chloramphenicol (CHL), sparfloxacin (SPX), doxycycline (DOX), and erythromycin (ERY).

Compound	MIC [mM] ATCC 13048	MIC [mM] CM 64	MIC [mM] Ea294	MIC [mM] Ea289
**29–36**	>2	>2	>1	>1
NAL	0.034	0.55 (16)*	4.4	>17.6
CHL	0.012	0.79 (66)	0.2	3.2
SPX	0.00015	0.0025 (17)	>2	>2
DOX	0.002	0.07 (35)	0.002	0.07
ERY	0.35	0.7 (2)	0.087	0.35
PAβN	5	5 (1)	0.0625	3.5

*MIC ratio: efflux overproducer/basal producer strains.

### Determination of the FIC Index

To determine the fractional inhibitory concentration index (FICi), a two dimensional checkerboard with twofold dilutions of each compound was set up for the study (Berenbaum, 1978; Allam et al., 2014). For the first clear well in each row of the microplate containing an antimicrobial agent, the FIC was calculated as follows: FIC of compound A (FIC A) = MIC of compound A in combination with B/MIC of compound A alone, and FIC of compound B (FIC B) = MIC of compound B in combination with A/MIC of compound B alone. The FICi was calculated as the sum of the FIC of each compound. The nature of the interaction was classified as follows: synergy FICi ≤ 0.5; additivity 0.5 < FICi ≤ 1; indifference 1 < FICi ≤ 2; and antagonism FICi > 2 (European Committee for Antimicrobial Susceptibility Testing (EUCAST) of the European Society of Clinical Microbiology and Infectious Diseases (ESCMID), 2000). For each combination, an isobologram, which graphically illustrates the interaction effect, was constructed: FIC A was showed in the abscissa and FIC B in the ordinate, the profile of the corresponding curve reflects the nature of the interaction.

## Results

### Antibacterial Activity

It is important that putative inhibitors that could be used as “adjuvant molecule” for antibiotics, do not display a high intrinsic antibacterial activity (Davin-Regli et al., 2008; Bolla et al., 2011). The determination of the antibacterial activity for each compound was performed and presented in **Table 1**. Many of the compounds did not inhibit the growth of bacteria even at the highest tested concentration as the previous generations of hydantoin derivatives (Handzlik et al., 2011). The majority of compounds exhibited a MIC > 2 mM in ATCC13048 and in CM64 strains and MIC ≥ 1 mM in Ea289 and Ea294.

### Influence on Antibiotic Susceptibility in ATCC 13048 and CM 64 Strains

#### Effect on Nalidixic Acid Susceptibility

**Table 2** presents the chemosensitizing effect of compounds on ATCC 13048 and CM 64 susceptibility to nalidixic acid (NAL). The gain on antibiotic activity (A) was calculated for each compound and each antibiotic. Regarding the CM 64 strain, a moderate or a weak effect was observed on the MIC when compounds were used at 0.0625 mM compared to PAβN. Compounds of generations IIIA (**29**–**32**) and IIIB (**33**–**36**) were used at a concentration of 0.0625 mM and 0.5 mM due to their higher MIC. A higher concentration of compounds of the generation IIIB did not improve the antibiotic activity whereas we observed a significant decrease of the antibiotic MIC, from 4- to 32-fold in CM 64 (see A_NAL_ in **Table 2**), in the case of compounds of generation IIIA (**29–32**). These compounds decreased the MIC of NAL from 8- to 16-fold comparing to the PAβN effect (64-fold) in the reference ATCC 13048 strain (see A_NAL_ in **Table 2**). In the case of PAβN and derivatives **32** the difference in activity in both tested strains was not significant (only a twofold stronger activity in the strain overexpressing the AcrAB pump than in the reference one) whereas compounds **29–31** showed a better action on antibiotic activity in the reference strain. It must be noted that the compound **32** used at increased concentration exhibits an activity profile similar to PAßN, which has been shown to be an efficient efflux inhibitor at low concentrations (Nikaido and Pagès, 2012; Misra et al., 2015).

**Table 2:** Effect of the hydantoin derivatives on the susceptibility level of *E. aerogenes* ATCC 13048 and CM 64 strains to nalidixic acid (NAL), chloramphenicol (CHL), and sparfloxacin (SPX).

Compound	Concentration [mM]	A _NAL_		A _CHL_		A _SPX_	
		ATCC 13048	CM 64	ATCC 13048	CM 64	ATCC 13048	CM 64
**29**	0.0625	1	1	1	1	2	2
**30**	“	2	2	2	1	1	1
**31**	“	2	2	2	1	1	2
**32**	“	2	4	1	1	1	2
**33**	“	1	1	1	1	1	1
**34**	“	1	0.5	0.5	1	0.5	1
**35**	“	1	1	0.5	1	0.5	0.5
**36**	“	1	1	1	1	0.5	0.5
**29**	0.5	8	4	2	4	4	2
**30**	“	8	4	4	4	8	4
**31**	“	16	4	4	4	4	4
**32**	“	16	32	4	32	8	8
**33**	“	2	2	1	2	2	1
**34**	“	2	1	1	1	2	2
**35**	“	2	1	2	1	2	1
**36**	“	2	1	2	1	2	1
**PAβN**	0.05	64	128	2	64	8	32

*“A” corresponds to the antibacterial activity gain obtained in the presence of the respective compound and evaluated for each antibiotic (A = MIC without compound/MIC with compound).*

#### Effect on Chloramphenicol Susceptibility

The results are presented in **Table 2**. In the case of the CM 64 strain, compounds tested at the concentration of 0.0625 mM showed a weak chemosensitizing effect on CHL antibacterial activity compared to PAβN. Compounds of generation IIIA (**29**–**32**) examined at a concentration of 0.5 mM decreased the chloramphenicol MIC from 2- to 4-fold in the reference strain and from 4- to 32-fold in the strain overexpressing efflux pump (see A_CHL_ in **Table 2**). We observed a noticeable increase in CHL susceptibility in the AcrAB overproducer CM 64, a 32-fold gain in the susceptibility with the compound **32** compared to compounds **29–31.** In this assay, the chemosensitizing effect of compound **32** can be compared to PAβN which was much more active in the strain overexpressing the AcrAB pump than in the parental strain ATCC 13048.

#### Effect on Sparfloxacin Susceptibility

**Table 2** presents the effects of hydantoins on sparfloxacin (SPX) susceptibility. In CM 64, the chemosensitizing effect of low concentrated hydantoins on SPX was as weak as we observed in the case of CHL. Compounds of generation IIIA (**29**–**32**) tested at the highest concentration 0.5 mM caused a 4–8-fold decrease in the MIC in the ATCC 13048 strain and a 2–8-fold decrease in the MIC in CM 64 one. Comparing the results obtained for the active compounds (**29–32**) to the results obtained for PAβN (see A_SPX_ in **Table 2**) we observed that the action of hydantoins with SPX was less efficient than PAβN, which was more active in the CM64 strain overexpressing the AcrAB pump than in the reference strain ATCC13048. This may suggest a different conformational site for the two molecules, either for recognition or for binding step, in the AcrB monomer (Delmar et al., 2014; Du et al., 2014; Yamaguchi et al., 2015), inside the pump or a different mode of action on the resistance mechanism.

### Effect of Compounds 29–32 on the Resistance Level in MDR *E. aerogenes* Strains

In order to evaluate the chemosensitizing effect of the compounds **29**–**32** (the most effective molecules) on the MDR background, the Ea289 and Ea294 strains were assayed. It is important to mention that these strains contain various resistance mechanisms (Malléa et al., 1998; Pradel and Pagès, 2002) such as target mutations (e.g., mutations in QRDR region of gyrase that increase quinolone resistance) and expression of modifying enzymes (e.g., ß-lactamases that contribute to ß-lactam resistance). **Table 3** presents the activity gain parameter A tested with doxycycline (DOX), erythromycin (ERY), and nalidixic acid (NAL) in parental and AcrAB-derivative strain context. It is interesting to note that **32** exhibited significant restoring antibiotic activity with DOX in Ea289 and very weak action in Ea294 that is devoid of the AcrAB efflux pump components. In contrast, a weak chemosensitizing activity was observed with ERY, this effect could be caused by the presence of additional resistance mechanisms for the macrolide antibiotic class as previously reported (Chollet et al., 2004). Regarding the effect on NAL and sparfloxacin susceptibility, the mutations in the quinolone target (DNA gyrase) previously reported in Ea289, can explain the weak effect observed toward these strains (Pradel and Pagès, 2002; Kaščáková et al., 2012).

**Table 3:** Effect of the compounds of generation IIIA (**29**–**32**) depends on AcrAB context.

Compound	Ea294			Ea289			CM 64	
	A_DOX_	A_ERY_	A_NAL_	A_DOX_	A_ERY_	A_NAL_	A_DOX_	A_ERY_
**29**	2	1	2	8	1	1	8	1
**30**	4	4	4	16	1	2	8	1
**31**	4	8	8	16	2	2	8	1
**32**	2	2	4	32	4	>2	32	2

*“A” corresponds to the antibacterial activity gain obtained in the presence of the respective compound and evaluated for each antibiotic (A = MIC without compound/MIC with compound). DOX, doxycycline; ERY, erythromycin; NAL, nalidixic acid.*

### Determination of the FIC Index for Compound 32

**FIGURE 2:**
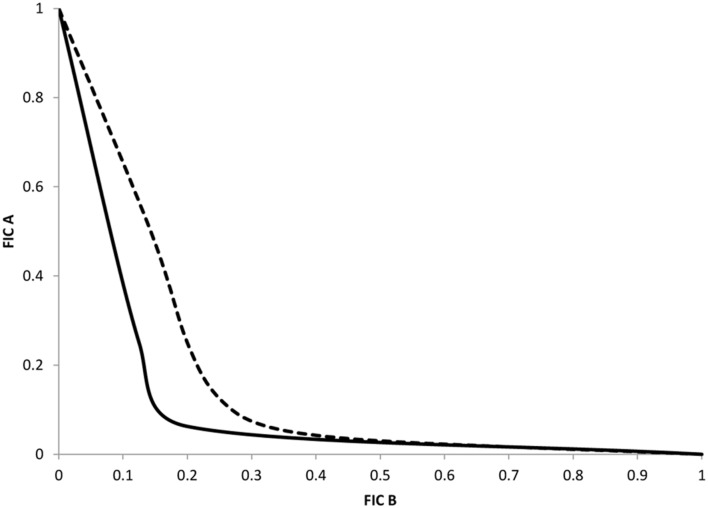
**Representative isobologram of interactions between compound 32 and nalidixic acid or chloramphenicol**. Nalidixic acid, solid line; chloramphenicol, dotted line. The axis numbers correspond to normalized FICs obtained with *Enterobacter aerogenes* CM64 strain.

To precise the type of interaction (synergistic, additive, or indifferent) between compound **32** and selected antibiotics, we carried out analysis based on the FICi as previously described. Combinations of compound **32** with NAL and CHL respectively were performed in CM 64 strain. This strain overexpresses the AcrAB pump and does not contain target mutation that can impair the effect of compounds on the restoration of antibiotic activity. The nature of the association was determined from the FICi average obtained from each combination and the representation was performed for each combination (**Figure 2**). In two cases, the isobologram curve obtained was concave indicating a synergy association between antibiotic and compound **32**. The synergistic association corresponds to an average of calculated FICi of about 0.32 for NAL and 0.44 for CHL respectively. These curves fitted well with the **Table 2** data.

## Discussion

The aim of this study was to identify compounds that are able to modulate the AcrAB pump activity and restore antibiotic activity on efflux producing strains.

The various molecules combined with NAL and CHL increased the susceptibility of the CM 64 strain and of the reference strain ATCC 13048 to antibiotics. On the one hand, we found compounds that showed stronger activity in the ATCC 13048 strain than in the CM 64 strain (**29–31** tested with NAL, **29, 30** tested with SPX). These findings could indicate in this case that tested compounds are not highly selective toward the AcrAB-TolC pump involved in the efflux of antibiotics. This suggests that they act not only on the basal AcrAB expressing strain but also that they may recognize other bacterial targets. In contrast, PAβN combined with CHL and SPX as well as the 3-aminobutyl-5-β-naphthylhydantoin **32** tested with CHL exhibited a difference between the AcrAB overproducer strain and the parental one. This time these results suggest a significant selectivity of chemosensitizers for the AcrAB pump expressed in bacterial strains. Taking into account the influence of concentration of 3-aminoalkyl-5-naphthylhydantoins (generation IIIA) on the antibiotic activity, we observed that the higher is the concentration, the stronger is the chemosensitizing effect, not only on the CM 64 strain but also on ATCC 13048. These outcomes could suggest additional mechanisms of action besides the effect on the AcrAB-TolC efflux pump.

The comparison of the results in Ea289 and Ea294 (**Table 3**) indicates the correlation of the restoring activity of antibiotics with the presence of the AcrAB efflux pump. They also illustrate the capability of compound **32** to increase the antibacterial effect of DOX in these strains. The low effect of **32** on the ERY susceptibility when assayed in Ea289 and Ea294 can be due to additional pump, other than AcrB, active in these clinical derivative strains capable to expel this class of drugs (Chollet et al., 2004).

The different structural features of tested compounds allow showing how the nature and the position of their diverse molecular fragments modulate on the one hand their selectivity regarding AcrAB pump and on the other hand their chemosensitizing activity. The tested compounds exhibiting the common hydantoin scaffold were modified using two of diverse substituents at position 5 as well as amine substituents and a selection of linkers to bind an amine to the hydantoin core. The location of an amine-alkyl fragment at position N1 or N3 to an aryl fragment at position 5 was modified for comparing with the activity of the first generations of hydantoin synthesized (Handzlik et al., 2011). In the case of **32**, the hydrophilic primary amine fragment is focused by terminate location at the end of longer 3-hydantoin substitution, opposite to the 5-β-nahthyl one.

The most active compounds (**29–32**) share the same pharmacophore profile than PAβN suggesting a similar physicochemical outline for an identical target. The amphiphilic nature of the Generation IIIA of optimized derivatives seems to be crucial to inhibit antibiotic resistance mediated by efflux pump and open new ways for generating original active compounds able to inhibit pump activity. With the recent published data regarding piperazine arylideneimidazolone derivatives as potential efflux inhibitors in *Escherichia coli* cells (Bohnert et al., 2016), the compound **32** characterized in this study will be used for pharmacophoric modulations in order to develop more potent inhibitors.

## Author Contributions

JH, KK-K, J-MP, and SA designed research; EO-M, JC, ES, and SA performed research; JS, GB, and J-MB contributed new reagents or analytic tools; EO-M, JH, KK-K, J-MP, and SA analyzed data; JH, KK-K, J-MP, and SA wrote the paper.

## Conflict of Interest Statement

The authors declare that the research was conducted in the absence of any commercial or financial relationships that could be construed as a potential conflict of interest.

## Footnotes

1 http://www.membranetransport.org/

2 http://www.tcdb.org/

3 http://clsi.org/
