# Supplementary material for: Efflux Pump Blockers in Gram-Negative Bacteria: The New Generation of Hydantoin Based-Modulators to Improve Antibiotic Activity
Source: Front Microbiol. 2016 May 3;7:622. doi: 10.3389/fmicb.2016.00622 (PMC4853399; doi:10.3389/fmicb.2016.00622)
Supplement: Supplementary file 1 [file Presentation_1.PDF]

Supplementary data

Efflux pump blockers in Gram-negative bacteria:
The new generation of hydantoin based modulators to improve antibiotic activity

Ewa Otrębska-Machaj [a, b], Jacqueline Chevalier [b], Jadwiga Handzlik [a], Ewa Szymańska [a], Jakub Schabikowski [a], Gérard Boyer [b], Jean -Michel Bolla [b], Katarzyna Kieć-Kononowicz [a], Jean -Marie Pagès [b*], Sandrine Alibert [b]

Synthesis Procedures

Syntheses of α-naphthyl compounds **29-31** were described earlier (Matys et al., 2015). Synthesis of the β-naphthyl analog 32 was performed in the same route as that of described for **30** (Matys et al., 2015) but using of 1-(naphthalen-2-yl)ethanone in place of 1-(naphthalen-1-yl)ethanone as a starting product.

3-(4-Aminobutyl)-5-methyl-5-(naphthalen-2-yl)imidazolidine-2,4-dione (32)

Yield 64 %; mp: 124-126ºC. Rf: 0.43 (7 : 3 : 0.1; DCM : MeOH : TEA).^{1}H-NMR for basic form of **32** (DMSO-d_6) δ [ppm]: 1.24-1.29 (m, 2H, alkyl), 1.48-1.53 (m, 2H, alkyl), 1.77 (s, 3H, -CH_3), 2.49 (t, *J*= 6.2 Hz, 2H, alkyl), 3.11 (br.s, 2H, -NH_2), 3.37 (t, *J*= 6.9 Hz, 2H, alkyl), 7.48-7.59 (m, 3H, Ar), 7.87-8.00 (m, 4H, Ar); ^{13}C-NMR (DMSO-d_6) δ [ppm]: 25.2, 25.5, 30.4, 38.3, 39.1, 39.4, 63.2, 124.0, 124.7, 127.0, 127.9, 128.6, 128.7, 132.8, 133.0, 137.6, 156.3, 175.8. LC/MS (m/z): 312.38 $[M+H]^+$ (96% purity)

Synthesis of compounds **33**-**36** was performed according to the Scheme 1. Commercial 4'-fluoroacetophenone (99% Aldrich) was a starting product (**37**) to obtain the intermediate 5-(4-fluorophenyl)-5-methylimidazolidine-2,4-dione **38** within Bucher-Bergs condensation (Ware, 1950). The oxiran intermediate **39** was obtained by Mitsunobu reaction with commercial racemic glycidol (96% Aldrich). Synthesis of intermediates **38** and **39** and final compound **36** (Goodson et al., 1960; Handzlik et al., 2014) were described elsewhere. The final compounds possess two chirality centres. As nonstereospecific Bucher-Bergs reaction was the synthesis method and the racemic reactant was used in the Mitsunobu one, the final compounds **33-36** were obtained in the form of a mixture of diastereomers, what was confirmed by melting points- and the spectral analysis performed.

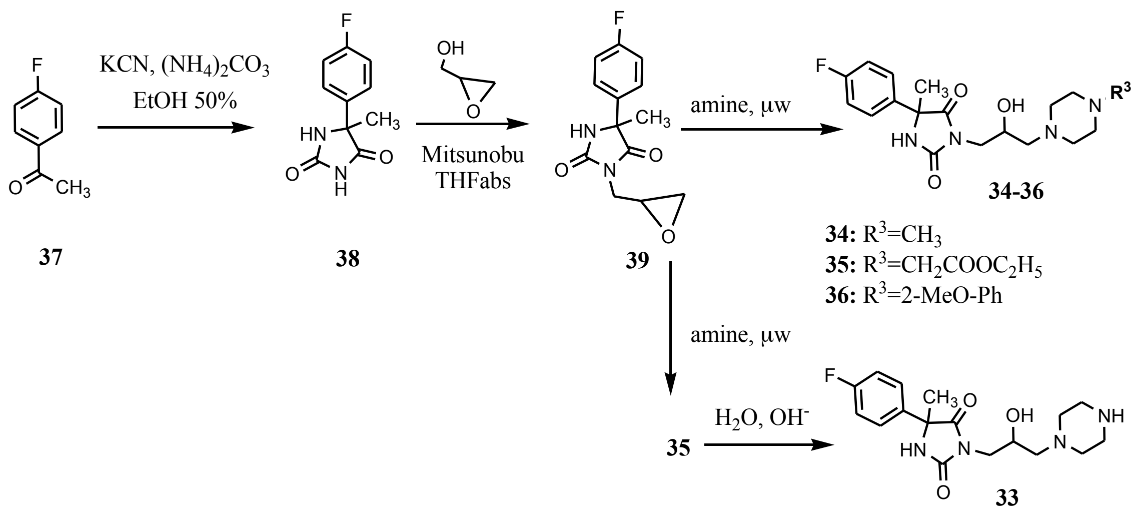

Scheme 1. Synthesis pathway for compounds of generation IIIB

Synthesis of 5-(4-fluorophenyl)-3-(2-hydroxy-3-(piperazin-1-yl)propyl)-5-methyl-imidazolidine-2,4-dione hydrochloride (33)

5-(4-Fluorophenyl)-5-methyl-3-(oxiran-2-ylmethyl)imidazolidine-2,4-dione **39** (5 mmol, 1.35 g) and 1-acetylpiperazine (5 mmol, 0.64 g) were dissolved in methylene chloride (5 mL). The solvent was evaporated. The residue was irradiated in household-microwave oven using an appropriate program of irradiation: 300 W (1 min), 450 W (1 min), 300 W (2 x 1 min). The obtained glue-residue of ester derivative was dissolved in 5 ml of EtOH, 3 ml of 15% HCl was added. The mixture was stirred and refluxed for 1.5 h, then 2 min with charcoal. The mixture was filtrated and neutralized with 25% ammonia and stored at 4ºC overnight, separated from the inorganic precipitate. The filtrate obtained was saturated with gaseous HCl to give yellow precipitate. The precipitate was crystalized with absolute EtOH (charcoal treatment) to give white powder of compound **33** in the form of a mixture of diastereomers.

Yield 19%; mp: 246-252˚C. Anal. Calcd. for $C_{17}H_{24}ClFN_4O_3$: C, 52.78; H, 6.25; N, 14.48; found: C, 52.59; H, 6.24; N, 14.46; ^{1}H-NMR (DMSO-d_6) δ [ppm]: 1.69 (br. s, 3H, 5-CH_3), 2.07 (br. s, 1H, 8-CH_a), 3.38 (s, 12H, 10,11,13,14-CH_2, 6-CH_2, 7-CH, 8-CH_b), 4.20 (br. s, 1H, 7-OH), 7.18-7.24 (t, *J*=8.84 Hz, 2H, 16,20-CH), 7.49-7.54 (dd def., 2H, 17,19-CH), 8.97 (br. s, 1H, 1-NH), 9.65 (br. s, 2H, 12-NH_2^+). IR (KBr) [cm-1]: 1601.59 (C=C; Ar), 1715.37 (C=O (4)), 1772.26 (C=O (2)), 2437.5 (NH^+), 2720.10 (CH; Aliph), 3002.62 (CH; Ar), 3408.57 (OH).

General procedure of synthesis of compounds 34 and 35

5-(4-Fluorophenyl)-5-methyl-3-(oxiran-2-ylmethyl)imidazolidine-2,4-dione **39** (3.5 mmol) and suitable piperazine (3.5 mmol) were dissolved in methylene chloride (5 mL). The solvent

was evaporated. The residue was irradiated in household-microwave oven using an appropriate program of irradiation: 300 W (2 min), 450 W (2 x 3 min), 300 W (2 x 1 min). The obtained glue-residue was purified with chromatography column (CH_2Cl_2/aceton/MeOH). The fractions containing the desirable product were collected and evaporated. The residue was dissolved in 99.8% EtOH (15 mL) and saturated with gaseous HCl to give precipitates of suitable hydrochlorides (**34** and **35)** after storing at 4°C overnight.

5-(4-Fluorophenyl)-3-(2-hydroxy-3-(4-methylpiperazin-1-yl)propyl)-5-methylimidazolidine-2,4-dione hydrochloride (34)

Yield 26%; mp: 239-245˚C. Anal. Calcd. for $C_{18}H_{26}ClFN_4O_3$: C, 53.93; H, 6.54; N, 13.98; found: C, 53.80; H, 6.67; N, 13.71; ^{1}H-NMR for basic form of **34** (DMSO-d_6) δ [ppm]: 1.64 (s, 3H, 5-CH_3), 2.08 (s, 3H, 12-CH_3), 2.10-2.25 (m, 10H: 8H, 10,11,13,14-CH_2, 8-CH_2), 3.14 (dd, 2H, 6- CH_2), 3.84 (s, 1H, 7-CH), 4.77 (br. s, 1H, 7-OH), 7.17-7.23 (m, 2H, 16,20-CH), 7.47- 7.53 (m, 2H, 17,19-CH), 8.86 (br.s, 1H, 1-NH). IR (KBr) [cm-1] for hydrochloride of **34**: 1598.70 (C=C; Ar), 1715.37 (C=O (4)), 1772.26 (C=O (2)), 2500.00 (NH+), 2929.34 (CH; Aliph), 2992.98 (CH; Ar), 3336.25 (OH).

Ethyl 2-(4-(3-(5-(4-fluorophenyl)-5-methyl-2,4-dioxoimidazolidin-3-yl)-2-hydroxypropyl) piperazin-1-yl)acetate hydrochloride (35)

Yield 20%; mp: 126-132˚C. Anal. Calcd. for $C_{21}H_{30}ClFN_4O_5$: C, 53.33; H, 6.39; N, 11.85; found: C, 52.99; H, 6.34; N, 11.57; ^{1}H-NMR for basic form of **35** (DMSO-d_6) δ [ppm]: 1.14 (t, J=7.00 Hz, 3H, C$H_3$$CH_2$), 1.64 (s, 3H, 5-$CH_3$), 2.19 (d, J=5.9 Hz, 2H, 8-CH_2), 2.34- 2.48 (m, 8H, 10,11,13,14-CH_2), 3.13 (s, 2H, 21-CH_2), 3.28-3.39 (m, 2H, 6-CH_2), 3.8- 3.85 (m 1H, 7-OH), 4.01 (q def. 2H, 23-CH_2), 4.78 (br. s, 1H, 7-OH), 7.17-7.23 (m, 2H, 16,20-CH), 7.47-7.52 (m, 2H, 17,19-CH), 8.85 (br. s, 1H, 1-NH). IR (KBr) [cm-1] for hydrochloride of **35**: 1600.63 (C=C; Ar), 1692.23 (C=O (4)), 1746.23 (C=O (2)), 2560.00 (NH+), 2943.80 (CH; Aliph), 3078.80 (CH; Ar), 3436.53 (OH).

Supplementary References

Goodson, L.H., Honigberg, I.L., Lehman, J.J., Burton, W.H. (1960). Potential Growth Antagonists. I. Hydantoins and Disubstituted Glycines. *J Org Chem.* 25: 1920-4

Handzlik, J., Bojarski, A. J., Satała, G., Kubacka, M., Sadek, B., Ashoor, A., et al. (2014). SAR-Studies on the importance of aromatic ring topologies in search for selective 5-HT_7 receptor ligands among phenylpiperazine hydantoin derivatives. *Eur. J. Med. Chem.* 78, 324-339.

Matys, A., Podlewska, S., Witek, K., Witek, J., Bojarski, A. J., Schabikowski, J., et al. (2015). Imidazolidine-4-one derivatives in the search for novel chemosensitizers of *Staphylococcus aureus* MRSA: Synthesis, biological evaluation and molecular modeling studies. *Eur. J. Med. Chem*. 101, 313-25.

Ware, E. (1950). The chemistry of hydantoins. *Chem. Rev*. 46, 403-470.
